# Network Pharmacology Prediction and Pharmacological Verification Mechanism of Yeju Jiangya Decoction on Hypertension

**DOI:** 10.1155/2021/5579129

**Published:** 2021-05-10

**Authors:** Ting Wang, Mao He, Yuzhong Du, Suhong Chen, Guiyuan Lv

**Affiliations:** ^1^College of Pharmaceutical Sciences, Zhejiang Chinese Medical University, Hangzhou 310053, China; ^2^School of Medicine, Taizhou University, Taizhou, Zhejiang 318000, China; ^3^Collaborative Innovation Center of Yangtze River Delta Region Green Pharmaceuticals, Zhejiang University of Technology, Hangzhou 310014, China

## Abstract

**Background:**

Yeju Jiangya decoction (CIF) is an herbal formula from traditional Chinese medicine (TCM) for the treatment of hypertension.

**Materials and Methods:**

Based on the analysis of network pharmacology, combined with in animal experiments, the network pharmacology was used to explore the potential proteins and mechanisms of CIF against hypertension. The bioactive compounds of CIF were screened by using the platform, and the targets of hypertension and CIF were collected. Then, the Kyoto Encyclopedia of Genes and Genomes (KEGG) and protein-protein interaction network (PPI) core targets were carried out, and the useful proteins were found by molecular docking technology. Finally, we used N-nitro-L-arginine (L-NNA) induced hypertension model rats to confirm the effect and mechanism of CIF on hypertension.

**Results:**

14 bioactive compounds of CIF passed the virtual screening criteria, and 178 overlapping targets were identified as core targets of CIF against hypertension. The CIF-related target network with 178 nodes and 344 edges is constructed. The topological results show that quercetin and luteolin are the key components in the network. The key targets NOS3 (nitric oxide synthase 3) and NOS2 (nitric oxide synthase 2) were screened by the protein-protein interaction network. The analysis of target protein pathway enrichment showed that the accumulation pathway is related to the vascular structure of CIF regulation of hypertension. Further verification based on molecular docking results showed that NOS3 had the good binding ability with quercetin and luteolin. On the other hand, NOS3 has an important relationship with the composition of blood vessels. Furthermore, the animal experiment indicated that after the L-NNA-induced hypertension rat model was established, CIF intervention was given by gavage for 3 weeks, and it can decrease serum concentrations of endothelin-1 (ET-1) and thromboxane B2 (TXB_2_), increase the expression of nitric oxide (NO) and prostacyclin 2 (PGI_2_), and improve renal, cardiac, and aortic lesions. At the same time, it can reduce blood pressure and shorten vertigo time. Western blot (WB) and immunohistochemistry (IHC) analyses indicated that CIF may downregulate the expression of NOS3, guanylyl cyclase-alpha 1 (GC-*α*1), guanylyl cyclase-alpha 2 (GC-*α*2), and protein kinase CGMP-dependent 1 (PRKG1). These results suggest that CIF may play an antihypertensive role by inhibiting the activation of the NOS3/PRKG1 pathway.

**Conclusions:**

The results of this study indicate that CIF has the ability to improve target organs, protect endothelial function, and reduce blood pressure and that CIF might be a potential therapeutic drug for the prevention of hypertension. It provides new insight into hypertension and the potential biological basis and mechanism for CIF clinical research.

## 1. Introduction

Hypertension is a common cardiovascular and cerebrovascular disease [1]. Based on the statistics, the prevalence of hypertension among adults in China was 5.1% in 1959, 19% in 2002, and 25.2% in 2012. According to the “Guidelines for the Prevention and Treatment of Hypertension in China” in 2018, the awareness rate, treatment rate, and control rate (crude rate) of hypertensive patients in China have increased significantly in recent years [2], but the overall level is still at a low level, reaching 51.6%, 45.8%, and 16.8%, respectively. The American Heart Association (AHA) redefined hypertension from 140/90 mmHg to 130/80 mmHg in the new edition of hypertension guidelines [3], further increasing the prevalence of hypertension. Therefore, the etiology and pathogenesis of hypertension and the development of therapeutic drugs are still important research subjects in the medical field [4, 5].

Traditional Chinese medicine (TCM) classifies hypertension as vertigo and headache [6]. The disease is mainly characterized by the deficiency of the upper and lower numbers of blood pressure, which is mixed with the deficiency and reality [7]. It is the hyperactivity of liver-fire and disturbance of wind-fire and wind-phlegm, while the deficiency of the liver and kidney leads to the deficiency of lower yuan in TCM. In the early stage of the lesion, the excess syndrome is the main factor, but in the late stage of the lesion, it becomes deficiency syndrome [8]. Syndrome differentiation can be roughly divided into four types: hyperactivity of liver-yang, phlegm turbidity obstructing orifice, deficiency of liver and kidney yin, phlegm, and blood stasis obstruction. According to the different syndromes, the treatment of hypertension in TCM is also different. In recent years, TCM has gradually become the main choice of new drugs for treating hypertension because of its various components, ways, and targets.

Yeju decoction (CIF) [9] is composed of traditional Chinese medicine Flos Chrysanthemi Indici, Fructus Ligustri Lucidi, and Angelicae Sinensis Radix. The chemical composition of Flos Chrysanthemi Indici includes sesquiterpenes, flavonoids, and phenolic compounds [10]. It has various functions such as clearing the heat and detoxification, soothing the wind, and calming the liver. In “Chinese Materia Medica,” Flos Chrysanthemi Indici is described as bitter, pungent, sexual flat, return to the lung and liver meridian [11] and can treat hypertension and other diseases. Modern pharmacological studies have also shown that Flos Chrysanthemi Indici can reduce blood pressure by inhibiting adrenaline and expanding the peripheral blood vessels [12–17]. Many studies have shown that Flos Chrysanthemi Indici can reduce blood pressure and anti-inflammation, and it has a certain role in the treatment of cardiovascular diseases and antiplatelet aggregation [18]. Previous studies also found that Flos Chrysanthemi Indici and its active ingredients, linarin [19]and luteolin [20], have significant antihypertensive effects in spontaneously hypertensive rats (SHRs) [21]. Jimaitong tablet, a Chinese patent medicine, is mainly composed of Flos Chrysanthemi Indici, and it is in the clinical trial stage. Therefore, according to the principles of TCM differentiation of symptoms and treatment and the compatibility of monarchs and ministers, this study regards CIF as a monarch medicine, a new extract prescription with different effects such as clearing the liver and purging fire, nourishing the liver and kidney, activating blood circulation, and removing blood stasis [18]. With the system characteristics of multicomponent, multichannel, and multitarget synergistic action of TCM extract prescription, it can be compared with a single prescription for the treatment of hyperactivity of liver-yang, deficiency of liver-kidney yin, and stagnation of phlegm and blood. In this study, we will use N-nitro-L-arginine (L-NNA) intraperitoneal injection to replicate hypertensive model rats and preliminarily evaluate the hypotensive effect of water extract of CIF using a noninvasive blood pressure system.

**Figure 1:**
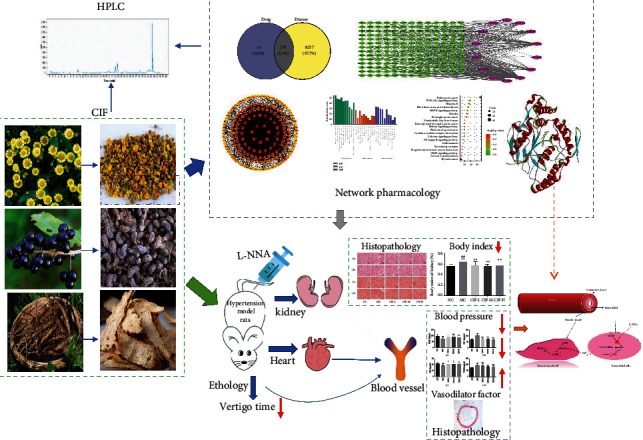
Whole study design.

Network pharmacology is a new method, initially used for drug discovery and development. With the rapid development of bioinformatics and systems biology [22], this method has the characteristics of multicomponent and multitarget [23]. In this study, we used network pharmacology analysis to investigate the mechanism of CIF on hypertension, and it further verified the effect of CIF in vivo (Figure 1).

## 2. Materials and Methods

### 2.1. Network Pharmacology Analysis

Yeju Jiangya decoction (CIF) consists of three kinds of traditional Chinese medicine, namely, Flos Chrysanthemi Indici, Fructus Ligustri Lucidi, and Angelicae Sinensis Radix.

The chemical components and related targets of the Flos Chrysanthemi Indici, Fructus Ligustri Lucidi, and Angelicae Sinensis Radix in CIF should be retrieved from the traditional Chinese medicine systems [24] pharmacology database and analysis platform (Tcmsp, http://tcmspw.com/tcmsp.php), and the rules are oral bioavailability (OB) ≥ 30% and drug-likeness (DL) ≥ 0.18. Then, the UniProt database (https://www.uniprot.org/) is used to query the gene name of the target protein [25]. Using “hypertension” as the keyword, the gene cards database and OMIM database were used to search and screen the disease target genes [25–27], and the duplicate targets in the search results were deleted to obtain the targets of hypertension. Then, the CIF target and hypertension target are mapped, and the Venny map (Venny 2.1) is drawn to get the intersection target of CIF and hypertension, which can be used as the potential target of CIF in the treatment of hypertension.

The chemical components and intersection targets of CIF were imported into Cytoscape 3.7.1 software to construct the active component target network, and the topological properties of the network were analyzed; the intersection targets were uploaded to STRING v11.0 database, the species was limited to “human,” the confidence was 0.4, the free nodes were deleted, and the protein-protein interaction (PPI) network was constructed [28–32].

Furthermore, the Metascape database was used for the gene biological process (GO) enrichment analysis and signal pathway (KEGG) enrichment analysis of CIF antihypertensive targets, and the bioinformatics platform was used for visualization analysis of biological functions and pathways. *P* value < 0.01 was used as the basis for screening biological functions and signal pathways of CIF antihypertensive targets.

For component target molecular docking, using molecular docking technology to study the active components of CIF and its related targets in the treatment of hypertension can explain the mechanism of action and binding activity of active components and target proteins to a certain extent. The structure of compounds in the SDF format was downloaded from PubChem (https://pubchem.ncbi.nlm.nih.gov/), and it was transformed into mol2 format file by Chem 3D software. The PDB format structures of proteins were downloaded from the RCSB database (https://http://www.rcsb.org/). The solvent molecules and ligands were removed by PyMOL software, and AutoDock tool 1.5.6 was used. Software hydrogenation, calculation of charge, and distribution of atom type are saved in pdbqt format. Finally, we run AutoDock Vina 1.1.2 for molecular docking and use discovery studio 2020 to visually analyze the docking conformation.

### 2.2. Chemicals and Reagents

L-NNA was purchased from Shanghai Yuanye Biological Co., Ltd. (Shanghai, China). Hematoxylin and eosin staining (H&E) dyeing solution was purchased from Nanjing Jiancheng Institute of Bioengineering (Nanjing, China). ET-1 Kit, NO Kit, TXB_2_ Kit, PGI_2_ Kit, and guanosine 3′, 5′-cyclic monophosphate (cGMP) were purchased from Shanghai Yuanye Biotechnology Co., Ltd. (Shanghai, China). DAB kit (ab64238) purchased from Abcam Company (Cambridge, USA), improved sodium citrate antigen repair solution was purchased from Biyuntian Biotechnology Research Institute (Shanghai, China), rabbit two-step immunohistochemical kit was purchased from Zhongshan Jinqiao (Beijing, China), and PBS phosphate buffer powder was purchased from Solarbio Co., Ltd. (Beijing, China). Endothelial nitric oxide synthase (eNOS/NOS3, 32027) was purchased from Cell Signaling Technology (Danvers, USA). Anti-guanylyl cyclase-alpha 1 (GC-*α*1, ab50358) antibody and anti-guanylyl cyclase-alpha 2 (GC-a2, ab42108) antibody were purchased from Abcam Company (Cambridge, England). Protein kinase CGMP-dependent 1 antibody (PRKG1, DF7018) was purchased from Affinity (Los Angeles, America). *ß*-Actin antibody was purchased from Hua‘an Biotechnology Co., Ltd. (Hangzhou, China). Chemical reference materials including acid, hyperoside, isoquercitrin, quercitrin, linarin, quercetin, and luteolin were purchased from Yuanye Biotechnology Co., Ltd. (Shanghai, China).

CIF was from traditional Chinese medicine. *Flos Chrysanthemi Indici*, *Fructus Ligustri Lucidi*, and Angelicae sinensis Radix (Zhejiang University of traditional Chinese medicine decoction pieces Co., Ltd) were boiled in water 10 times, concentrated, and stored at 4°C until use.

### 2.3. Animal Treatment

Forty male SD rats (NO.201803602) were obtained from the Animal Supply Center of Suzhou New Pharmaceutical Research Center Co., Ltd. (Suzhou, China). All the animal experiments involved in the experiment are by moral and legal requirements, and the animal operations were following the Guidelines of Care and Use of Laboratory Animals published by the Zhejiang province. All the animals were hosted at room temperature (20–24°C) and relative humidity (50–70%) on a 12-hour light/dark cycle environment, and they have free access to standard rodent food and water.

After 7 days of acclimation to the laboratory, 40 rats were randomized into five groups (*n* = 8): normal control group (NC), model control group (MC), low Yeju Jiangya Decoction group (CIF-L), middle Yeju Jiangya Decoction group (CIF-M), and high Yeju Jiangya Decoction group (CIF-H). The normal control group was fed a basic diet and water, while the other 32 rats were orally intraperitoneal injection L-NNA (7.625 mg/kg a day) for 4 weeks to replicate animal models of hypertension. Then, each CIF group was given intragastrically daily at the doses of 1.75 g/kg, 3.50 g/kg, and 5.25 g/kg (calculated by crude medicine) for 3 weeks, while the NC and MC groups received an equal volume of water. All animals were given free access to food and water during the experimental period. We set up multiple positive drug groups, but to highlight the study of CIF, so no data are displayed.

### 2.4. HPLC Analysis

CIF was analyzed with HPLC-DAD. The analysis could simply describe that the sample was through a 0.22 m membrane filter before put into the equipment. During the research of CIF, samples were collected and analyzed by HPLC using the Shimadzu LC-16 on an Agilent ODS (4.6*∗*150 mm, 4 um) at 30°C. The solvent system was acetonitrile (A) and 0.5% formic acid aqueous solution (B*∗*). The gradient condition was as follows: 0–6 min (90%B), 6–15 min (90% B–86.5% B), 15–21 min (86.5% B–79% B), 21–30 min (79% B–75% B), and 30–60 min (75% B–0% B). The following rate was 1 mL/min, and the DAD detector was set at 360 nm.

### 2.5. Blood Pressure Measurement

To further stimulate the state of human blood pressure measurement, we used the blood pressure measured by BP-2010AUL (Ruanlong Co., Ltd., Beijing, China) to the noninvasive blood pressure measurement system in rats. Use the tail of the rat to put in the sensor of the blood pressure meter to measure the blood pressure systolic blood pressure (SBP), diastolic blood pressure (DBP), and mean blood pressure (MBP).

### 2.6. Behavior and Signs Index

Vertigo time determination: the rats were placed on the balance rotator, rotated at 500 r/min for 1 minute, then removed, and placed on a high platform to see if the rats could maintain balance and not fall and recorded the time needed for the rats to return to the normal state.

### 2.7. Measurement of Organ Coefficient

After the last administration, rats were fasted for 12 hours, weighed their bodyweight, and rats were euthanized by excessive intravenous injection of an overdose of pentobarbital (100 mg/kg) [33], and then, 5 mL of venous blood drawn from their veins was collected for subsequent experiments. After execution, the kidney and heart were separated, the total weight of the kidney was weighed, and the organ index was calculated. Organ index (mg/g) = organ weight/bodyweight.

### 2.8. Determination of Vasodilator Factor

Used from venous blood drawn and centrifuged at 3,500 r/min for 10 minutes and stored at –80°C. Serum was separated to determine ET-1, NO, TXB_2_, and PGI_2_ activities by Power-wave 340 (BioTek, USA) for ELISA kits.

### 2.9. Histopathology

First, the samples of organs were quickly dissected. The aorta, kidney, and heart were removed and fixed in neutral formaldehyde solution. Second, after soaking for 36 hours, the organ was dehydrated and embedded in tissue wax block, and then, 4 mm thick sections were stained with hematoxylin and eosin. Finally, sections were observed under the microscope.

### 2.10. Immunohistochemistry Observation

For IHC, mouse and rabbit-specific HRP/DAB (ABC) detection IHC kits were used for the development of the reaction of NOS3 (1 : 150), GC-*α*1 (1 : 200), GC-*α*2 (1 : 200), and PRKG1 (1 : 100) in the aorta, and observation was performed using a biological microscope (Motic BA410).

### 2.11. Determination of cGMP

After dissecting the samples aorta, using diluted 1 : 10 with physiological saline to prepare a homogenate, centrifuged at 3500r/min for 10 min, the supernatant was taken to determine cGMP for ELISA kit.

### 2.12. Western Blot Analysis

Aorta tissues were mechanically lysed in liquid nitrogen and protein extraction. The protein concentration was detected with a BCA protein assay kit. After sample protein was added to 5x loading buffer and protein at 95°C, an equivalent amount of total protein of samples was separated by SDS-PAGE at room temperature and electrotransfer onto a PVDF membrane. Membranes were blocked with 5% powdered milk for 2 h at room temperature and incubated overnight at 4°C with corresponding first antibody (solute in primary antibody dilution buffer) against the NOS3 (1 : 1000), GC-*α*1 (1 : 1000), GC-*α*2 (1 : 700), PRKG1 (1 : 750), and *ß*-actin (1 : 1000) were used as a loading control. After washed three times for 5 min each in PBST, membranes were incubated with the appropriate secondary antibodies HRP-conjugated goat anti-rabbit IgG (*H* + *L*) for 2 h at room temperature and washed again. The blotted protein bands were detected by the assay kit, and the protein expression levels were calculated to Image J.

### 2.13. Statistical Analysis

All values are expressed as mean ± standard deviation and subjected to one-way analysis of variance (ANOVA) by using SPSS 19.0 for windows. LSD *t*-tests were applied when homogeneity of variance assumptions was satisfied; otherwise, the Dunnett test was used. The value of *P* < 0.05 was considered statistically significant.

## 3. Results

### 3.1. Network Pharmacology Analysis of CIF against Hypertension

#### 3.1.1. Screening of Active Components and Targets in CIF

Search Tcmsp database for all the active ingredients and Flos Chrysanthemi Indici, Fructus Ligustri Lucidi, and Angelicae Sinensis Radix related targets, and add gene names to the selected targets. According to the parameters of OB and DL, bioactive compounds of CIF, 2 active components of Angelicae Sinensis Radix, 12 active components of Flos Chrysanthemi Indici, and 13 active components of Fructus Ligustri Lucidi, were obtained. A total of 617 target genes were obtained, including 69 target genes of Angelicae Sinensis, 290 target genes of Flos Chrysanthemi Indici, and 352 target genes of Fructus Ligustri Lucidi.

**Figure 2:**
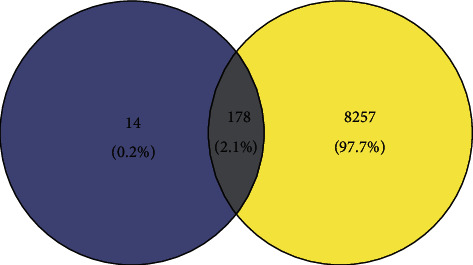
The target of CIF and hypertension. The purple circle indicates the CIF, and the yellow circle indicates the disease.

With “hypertension” as the keyword, we searched the GeneCards database and OMIM database to get 8435 targets, 23 targets, 8435 disease-related targets after combined weight removal, and 178 intersection targets after the intersection of the two, which may be the potential targets of CIF in the treatment of high blood pressure in Figure 2.

#### 3.1.2. Construction of the Active Ingredient Target Network

**Figure 3:**
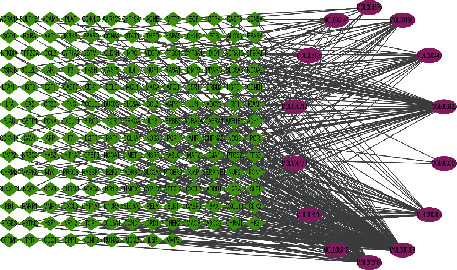
Active ingredient-target network. The green squares indicate the target, and the purple squares indicate the compounds.

The active component target network of CIF consists of 14 compound nodes, 178 target nodes, and 344 edges. As shown in Figure 3, CIF has the characteristics of multicomponent and multitarget in the treatment of hypertension. The same active component can act on different targets, and the same target can also correspond to different active components. The topological results showed that quercetin (MOL000098) and luteolin (MOL000006) were the key components in the network.

#### 3.1.3. Construction of the PPI Network

**Figure 4:**
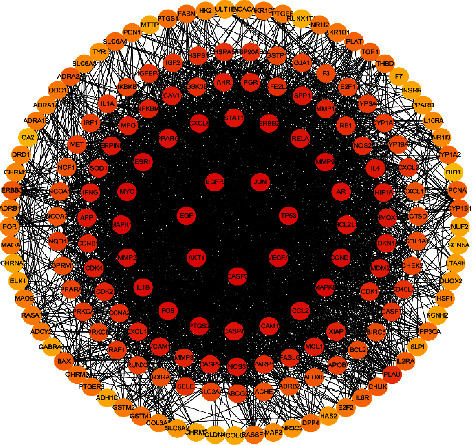
PPI network of CIF for hypertension. Orange, yellow, and red circles indicate the proteins. The black lines represent the nodes associated through protein-protein interactions.

The PPI network of target protein was constructed in the STRING v11.0 database. The species was set as “Homo sapiens,” the minimum interaction threshold was set as medium confidence, and the “medium confidence” was set as 0.4. The rest parameters remained the default settings. NOS3, NOS2, AKT1, TP53, and VEGFA were the key targets of CIF in the treatment of hypertension as shown in Figure 4.

#### 3.1.4. GO Function Enrichment Analysis and KEGG Pathway Enrichment Analysis

**Figure 5:**
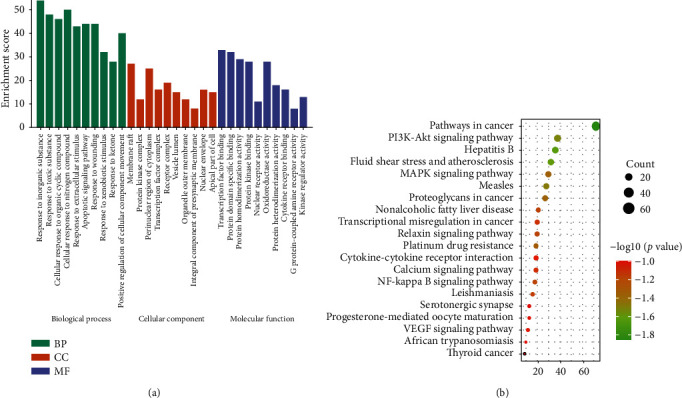
Functional analysis of CIF against hypertension: (a) GO analysis of key targets. (b) KEGG analysis of core targets. The color of circles represents the *Q* value, and the size of circles represents the count.

The 178 proteins involved in the compound name component target network were analyzed by GO classification and KEGG pathway enrichment analysis, and *P* < 0.01 was used as the screening condition to obtain the biological process and metabolic pathway with more enriched genes. In GO enrichment analysis, 60 GO items and 20 biological process related items were identified, involving reactions to inorganic substances, toxic substances, cells to organic compounds, cells to nitrogen compounds, cells to extracellular stimulation, and molecular function related items. There are 20 items related to transcription factor binding, protein domain specific binding, protein homodimeric activity, protein kinase binding, and nuclear receptor activity. There are 20 items related to cell composition, including membrane raft, protein kinase complex, cytoplasmic perinuclear region, transcription factor complex, and receptor complex as shown in Figure 5(a).

The results of KEGG pathway enrichment analysis of CIF mainly involved vascular related pathways: cancer pathway, hepatitis B, fluid shear stress and atherosclerosis, PI3K Akt signaling pathway, measles, proteoglycans in cancer, MAPK signaling pathway, platinum resistance, relaxin signaling pathway, and NF-*κ*B signaling pathway, including 20 important pathways as shown in Figure 5(b).

#### 3.1.5. Molecular Docking Verification of Core Components in CIF

Through Internet pharmacology, the key targets, NOS3 and NOS2, which were selected from the protein interaction network of quercetin and luteolin, which ranked the top two in the network topology analysis, were docking one by one as shown in Table 1. It is generally believed that the lower the binding energy is, the more stable the binding between ligand and receptor is; ≤ 5.0 kcal/mol indicates that they can bind, and ≤ 7.0 kcal/mol indicates that they have the better binding ability. Molecular docking results showed that NOS3 and NOS2 had the good binding ability with quercetin and luteolin.

#### 3.1.6. Analysis of Molecular Docking Mode

**Figure 6:**
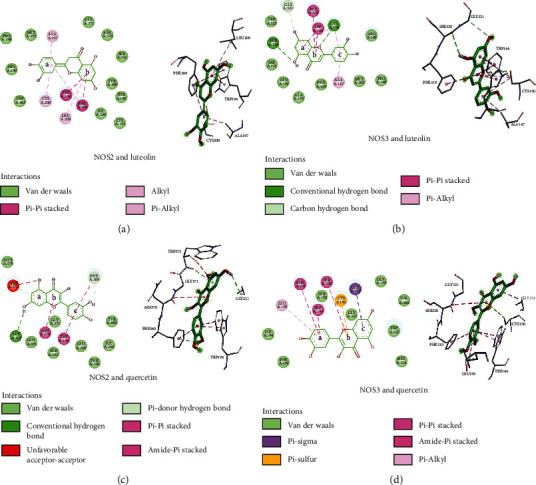
Interaction diagram of chemical composition and protein. (a) NOS2 and luteolin; (b) NOS3 and luteolin; (c) NOS2 and quercetin; (d) NOS3 and quercetin.

To further clarify, we used NOS3 and NOS2 proteins to dock with quercetin and luteolin as shown in Figure 6. When luteolin docks with protein NOS3, ring A formed a *π*-*π* stacking effect with trp144, hydrogen bond with gly321 and ser320, hydrophobic interaction with cys150; ring B formed a *π*-*π* stacking effect with trp144 and phe319, hydrophobic interaction, and hydrogen bond with cys150; and ring C formed hydrophobic interaction with cys150 and ala147. When luteolin interacts with protein NOS2, ring A forms hydrophobic interaction with ala197, cys200, and phe369, and ring B forms hydrophobic interaction with leu209 and a *π*-*π* stacking effect with trp194 and phe369. Quercetin docking with NOS2 showed that A ring formed a hydrogen bond with trp372 and gly202, B ring formed the amide-*π* stacking effect with asn370, and C ring formed a *π*-*π* stacking effect with trp194 and phe369 and formed a hydrogen bond with phe369. Quercetin docking with NOS3 showed that, A ring, on the one hand, with leu159 formed hydrophobic interaction, on the other hand, it formed a *π*-*π* stacking effect with phe319 and trp144. Its B ring interacts with ser320 to form the amide-*π* stacking effect and cys150 to form sulfur-*π* interaction, and C ring interacts with gly152 to form *π*-sigma interaction.

### 3.2. HPLC Analysis of CIF

**Figure 7:**
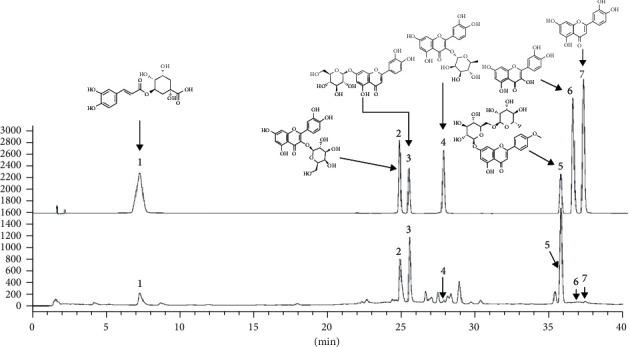
Representative HPLC-based chemoprofiles of CIF samples. The structures of analytes, which are labelled as follows: (1) Chlorogenic acid, (2) hyperoside, (3) isoquercitrin, (4) quercitrin, (5) linarin, (6) quercetin, and (7) luteolin.

HPLC was used to identify the main compounds in CIF. In addition to the luteolin and quercetin were identified in network pharmacology, and we also found that chlorogenic acid, hyperoside, isoquercetin, quercitrin, and linarin may contain this component through literature review. Seven chemical components of CIF were determined according to the spectrogram and retention time of reference materials as shown in Figure 7.

### 3.3. Blood Pressure Analysis of CIF against Hypertension

**Figure 8:**
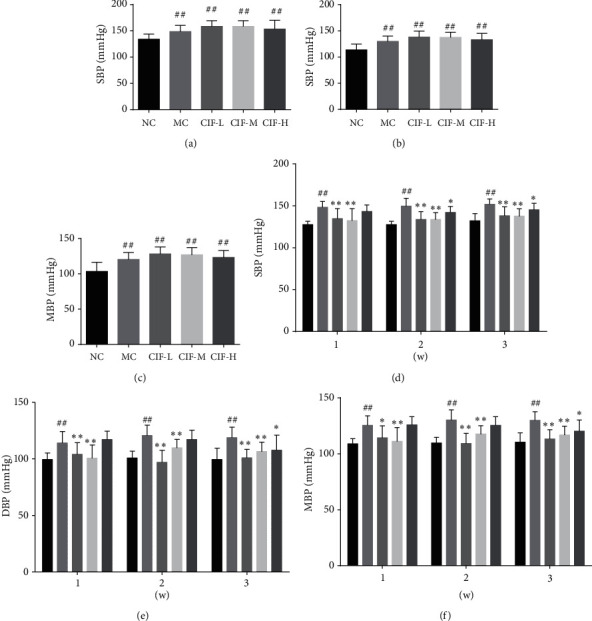
CIF used to hypertensive modern rats (*n* = 8) about blood pressure. (a–c) Blood pressure indexes of rats after injection of L-NNA for 4 weeks. (d–f) When the hypertension model is successful, after CIF administration, the blood pressure of rats changed for 1^st^ week, 2^nd^ week, and 3^rd^ week. The data were expressed as mean ± SD. ^#^*P* < 0.05 and ^##^*P* < 0.01 compared with the normal control group; ^*∗*^*P* < 0.05 and ^*∗*^, ^*∗*^*P* < 0.01 compared with the model control group. SBP, systolic blood pressure; DBP, diastolic blood pressure; MBP, mean blood pressure.

Increased blood pressure is the main manifestation of hypertension, so measure SBP, DBP, and MBP to evaluate blood pressure. As shown in Figures 8(a)–8(c), after the establishment of the model, compared with the normal control group, the blood pressure of the other groups increased significantly (*P* < 0.01), which indicated that the model was successful in hypertensive modern rats. As shown in Figures 8(d)–8(f), compared with the MC group, SBP, DBP, and MBP of blood pressure in rats decreased after 1 week of administration in the CIF-L, CIF-M, and CIF-H groups (*P* < 0.01, 0.05). Then, SBP, DBP, and MBP of blood pressure rats decreased after 1-2 weeks of administration in CIF-L and CIF-M groups (*P* < 0.01) and that of rats in the CIF-H group decreased significantly (*P* < 0.05). Three weeks later, the SBP of blood pressure in rats of the CIF-L group and CIF-M group decreased significantly (*P* < 0.05). SBP, DBP, and MBP of blood pressure in rats in the CIF-L group, CIF-M group, and CIF-H group were all decreased (*P* < 0.01, 0.05).

### 3.4. Organ Coefficient and Behavior Analysis of CIF against Hypertension

**Figure 9:**
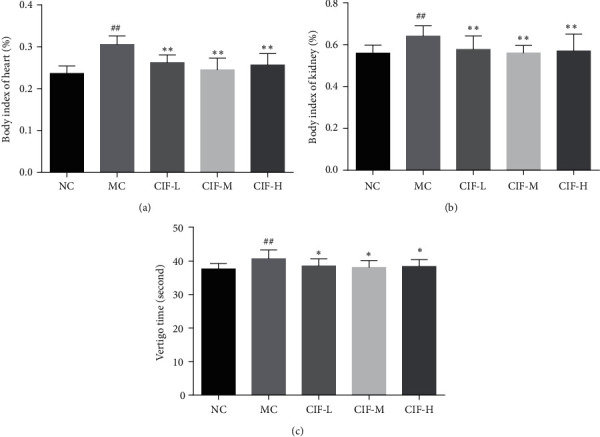
CIF to hypertensive modern rats (*n* = 8) about organ coefficient and vertigo times. (a) The body index of the heart; (b) the body index of the kidney; (c) the vertigo times. The data were expressed as mean ± SD. ^#^*P* < 0.05 and ^##^*P* < 0.01 compared with the normal control group; *∗*, *P* < 0.05 and *∗*, *∗*, *P* < 0.01 compared with the model control group.

Hypertension is generally accompanied by damage to target organs. As a cardiovascular disease, hypertension has a greater impact on the heart and kidneys. These two organs are selected for organ index testing. As shown in Figures 9(a) and 9(b), the heart index and kidney index of rats increased significantly (*P* < 0.01) after L-NNA administration, while CIF can reduce the heart index and kidney indices (*P* < 0.01).

After treatment with CIF, the “facial heat” and “dizziness” symptoms were recovered, suggesting that CIF may play a role in treating hypertension by alleviating the symptoms in rats. In the TCM argument, vertigo is the symptom of hypertension, and here, we simulate the situation of vertigo to see if CIF can improve the symptoms of vertigo in the rat model of hypertension. As shown in Figure 9(c), vertigo time was significantly higher compared to the NC group (*P* < 0.05) after 3 weeks of administration in the model group. The CIF-L group, CIF-M group, and CIF-H group compared with the MC group showed vertigo time of rats decreased (*P* < 0.05).

### 3.5. Observation of Pathological CIF against Hypertension

**Figure 10:**
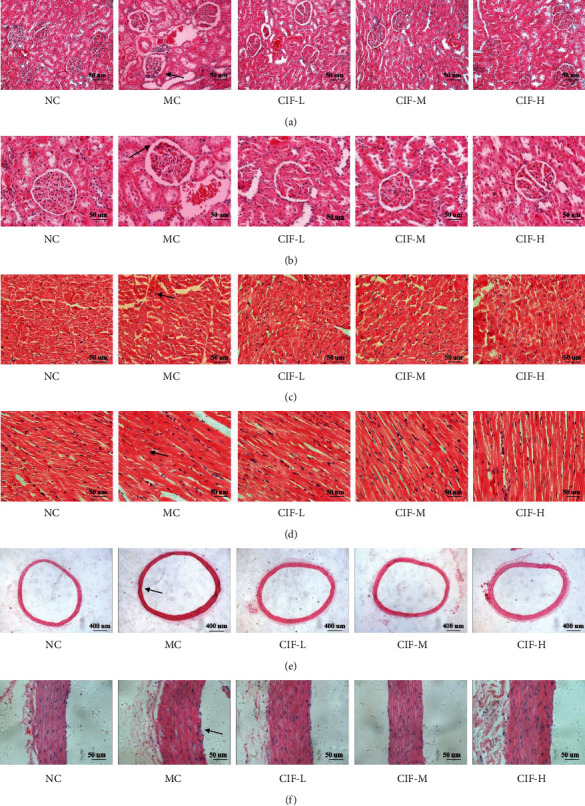
Observation of pathological effects of CIF on viscera for H&E staining. (a) Kidney (×200 magnification); (b) kidney (×400 magnification); (c) cardiac interstitium (×400 magnification); (d) ventricular myocardium (×400 magnification); (e) aorta (×40 magnification); (f) aorta (×400 magnification).

The lesions of the target organs can be observed by H&E staining. Especially, the pathological observation of the kidney, heart, and aorta related to hypertension. According to the results of kidney H&E staining, as shown in Figures 10(a) and 10(b), when compared with the normal control group, the renal tubules in the MC group showed signs of sclerosis and atrophy. Compared with the MC group, the degree of tubular sclerosis and atrophy in the CIF-L group, CIF-M group, and CIF-H group decreased in varying degrees.

From the myocardial interstitium in Figure 10(c), it can be seen that the model rats have a messy interstitium and large gaps. CIF can alleviate this situation and improve it. As shown in Figure 10(d), the model group has a disordered arrangement of myocardial fibers, enlarged gaps, and thickened parts of myocardial fibers. However, the myocardial stripes in the CIF group improved, and some of the myocardial fibers were arranged neatly, and the situation was improved.

Hypertension directly acts on blood vessels, and the damage of blood vessels will lead to an increase in blood pressure. In the blood vessels, blood exchange between the thoracic aorta and the heart plays an important role in raising blood pressure. The next results of the H&E staining of the aorta showed that compared with the normal control group, the intimal cells of the aorta in the MC group were damaged, with a small amount of exfoliation and uneven distribution, and the smooth muscle cells were thickened, arranged disorderly, and irregular, as shown in Figures 10(e) and 10(f). Compared with the MC group, the CIF could improve the injury of the intracellular membrane and inhibit the smooth muscle cells. Proliferation can alleviate the disorder and irregularity of cell arrangement.

### 3.6. Vasodilator Factor of CIF against Hypertension

Regulation of vascular endothelial tension depends on endothelial relaxation factors (EDRFs) and endothelial contraction factors (EDCFs). EDRFs include NO, PGI_2,_ and so on, among which NO is the most critical factor regulating vasodilation in vivo. [34] It can promote vasodilation and inhibit inflammation. EDCFs include ET-1 and TXB_2_. ET-1 can promote the proliferation of smooth muscle in vivo. It is the most persistent and powerful EDCFs at present. It can be bind to corresponding receptors which can promote prostaglandin synthesis and inhibit NO production [35]. EDRFs and EDCFs antagonize each other and play an important role in maintaining the balance of vasoconstriction and diastole.

**Figure 11:**
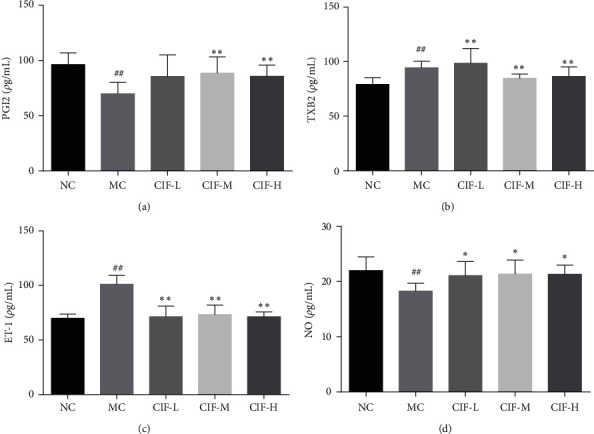
Effect of CIF on the vasodilator factor about hypertensive modern rats (*n* = 8) about ELISA kit. (a) TXB_2_, (b) PGI_2_, (c) ET-1, and (d) NO. The data were expressed as mean ± SD. ^#^*P* < 0.05 and ^##^*P* < 0.01 compared with the normal control group; *∗P* < 0.05 and *∗*, *∗P* < 0.01 compared with the model control group.

As shown in Figures 11(a) -11(d), the level of serum ET-1 and TXB_2_ in the model group was significantly increased compared to the normal control group (*P* < 0.01), while the level of serum NO and PGI_2_ were reduced (*P* < 0.01, 0.05); it indicated that the model was successful for promoting the vasoconstrictor factor of the blood vessel in hypertension rats. Compared with the model control group, after oral administration with Flos Chrysanthemi Indici extract, the results showed the level of serum ET-1 and TXB_2_ in CIF-H was significantly reduced (*P* < 0.01, 0.05) and the level of serum NO and PGI_2_ was increased (*P* < 0.01, 0.05).

### 3.7. NOS3 of CIF against Hypertension

**Figure 12:**
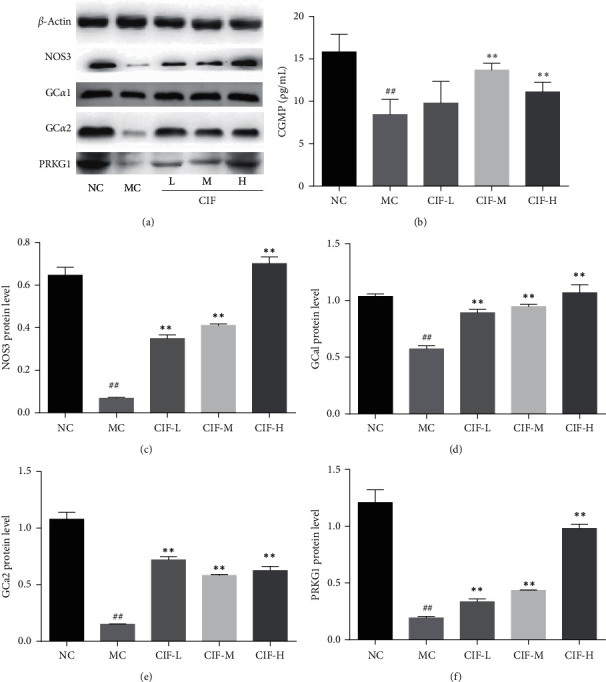
Effect of CIF on NOS3/GC*α*1/cGMP/PRKG1. (a) Representative results of NOS3/sGC/PRKG1 in Western blot; (b) cGMP in the aortic homogenate for ELISA; (c–f) protein level of NOS3, GC-*α*1, NOS3, GC-*α*2, and PRKG1. The data were expressed as mean ± SD. ^#^*P* < 0.05 and ^##^*P* < 0.01 compared with the normal control group; *∗*, *P* < 0.05 and *∗*, *∗*, *P* < 0.01 compared with the model control group.

According to the results of network pharmacology analysis and literature review, we found that NOS protein plays a key role. NOS is the key regulator of intravascular which regulates vascular tension by inhibiting smooth muscle contraction and platelet aggregation [36]. NO and L-citrulline are produced by NOS using L-arginine as substrate and oxygen. NOS can also produce oxidation products, such as NOS1 (nNOS), NOS2 (iNOS), and NOS3 (eNOS). Our results revealed CIF was inhibited in the NOS3/GC-*α*1/cGMP/PRKG1 and the aorta tissues of L-NNA injected rats as shown in Figure 12. Because cGMP has no antibody, it is found that the expression of CIF-M and CIF-H in serum is increased compared with the MC group. The expression of NOS3, GC-*α*1, GC-*α*2, and PRKG1 in the aorta and the content of cGMP in the aortic homogenate of model rats were detected. The expression of NOS3, GC-*α*1, GC-*α*2, and PRKG1 in the aorta and the content of cGMP in aorta homogenate of rats in each group increased after oral administration of CIF. At the same time, combined with the results of serum NO level determination, it is indicated that CIF can increase the expression of NOS3 in vascular endothelial cells, increase NO production, and activate the downstream GCa/cGMP/PRKG1 signaling pathway, thus causing vasodilation and lowering blood pressure.

**Figure 13:**
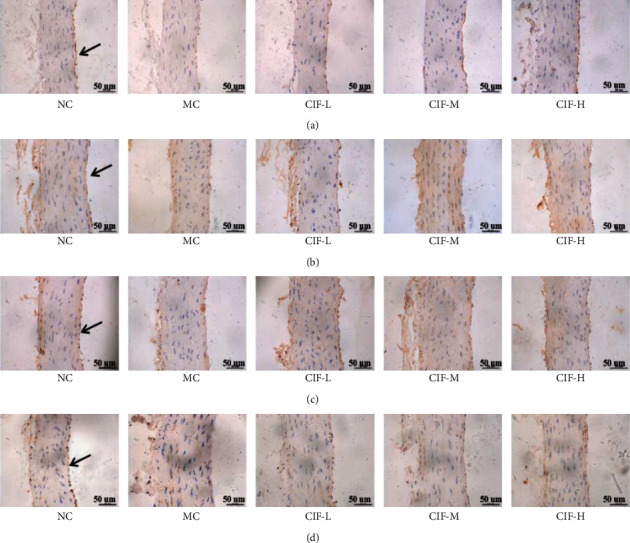
CIF on the protein expressions of NOS3GC-*α*/cGMP/PRKG1 in immunohistochemical IHC to observe myocardial histopathological changes of aorta (×200 magnification). (a) NOS3; (b) GCal; (c) GCa2; (d) PRKG1.

As the immunohistochemical results showed NOS3 staining was brown granular, mainly distributed in the endothelial cell membrane and interstitium of vascular endothelial cells. Compared with the normal control group, the endothelial cells of the aorta in the MC group were partially exfoliated, and the brown granular substances were significantly reduced. The GC-*α*1 staining was brown granular, mainly distributed in the interstitial cells, the brown granular substances in smooth muscle cells and endothelial cells of rats in the MC group decreased, and the expression of the brown-yellow substance increased in all groups of all CIF groups. The next is GC-*α*2 on the aorta; it was brown granular, mainly distributed in the interstitium that the brown granular substances in smooth muscle cells and endothelial cells of rats in the MC group were significantly reduced and the expression of the brown substance of rats in each group of CIF groups. About CGMP upstream of the NO pathway, it was also significantly decreased in the aortic homogenate. Finally, results showed that PRKG1 staining was brown granular, mainly distributed in the interstitium. The brown granular substances in aortic smooth muscle cells and endothelial cells of rats in the L-NNA model group were significantly reduced compared with the normal control group; compared with the MC group, the expression of brown substances in each group of CIF groups was increased in varying degrees as shown in Figure 13. This result suggests that CIF might regulate nitric oxide elevation in the aorta through the inhibition of the NOS3/PRKG1 pathway.

## 4. Discussion

In the present study, we used a network pharmacology approach to reveal the potential mechanisms of CIF against hypertension at a system level. Compound-target network construction indicated that luteolin and quercetin were the most important compounds in the network. Through network pharmacology KEGG enrichment and protein docking, we found that hypertension is closely related to blood vessels, and we found that NOS plays a key role in blood vessels, so we conducted related research. However, traditional Chinese medicine has multiple targets and functions.

CIF is the core target of hypertension in the aspect of blood vessels. Vascular endothelial cells (VEC) dysfunction is one of the important mechanisms of hypertension. VEC is a layer of monolayer active cells that continuously cover the surface of the whole vascular lumen. It can provide a smooth surface for blood flow and regulate vascular tension, and it has been also used to maintain the normal flow of blood. Regulation of vascular endothelial tension depends on NO in endothelial relaxing factors (EDRFs) which can promote vasodilation [34, 37], while PGI2 has a strong vasodilator effect. L-NNA is commonly used as a model drug for hypertension [38–46] because the occurrence of hypertension is generally accompanied by vascular endothelial dysfunction [47], which is mainly manifested by the reduction of the endothelial vasodilator factor (EDF) production and release. The secretion of NO in VEC occurs through autocrine, endocrine, and paracrine pathways. These vasoactive substances play a role in regulating vasoconstriction to maintain the steady state of vascular function [48]. Furthermore, the results of this part of the experiment showed that the levels of NO and PGI2 in the serum of model rats decreased, while the levels of ET-1 and TXB2 increased. After a period of intragastric administration of CIF, the levels of NO and PGI2 increased, while the levels of ET-1 and TXB2 decreased [49]. And also in this study, the rats developed symptoms such as a decrease in heart rate and an increase in blood pressure after the intraperitoneal injection of L-NNA, renal tubular atrophy, and sclerosis, suggesting that the model was successfully replicated. In this study, through pathological observation, the heart and kidney lesions appeared in the L-NNA model and the heart, consistent with the results reported in the literature [50–52].

When hypertension occurs, the tension of vascular endothelium will increase while stimulating endothelial cells to produce and release EDCFs and various growth factors. The increase in vascular wall tension cannot match the increase in blood flow under hypertension, while the lack of vascular smooth muscle cells (VSMC) will increase collagen fibers, thicken vascular walls, and decrease vascular elasticity, leading to vascular endothelial function injury [53, 54]. During this process, peripheral resistance increases continuously, vascular contraction and diastolic dysfunction occur, and eventually, VEC damage is aggravated by feedback. H&E results showed that the endothelial cells of the model group were damaged; a small amount of endothelium was exfoliated, and the nuclei were unevenly distributed. The smooth mesangial muscle cells were thickened and arranged disorderly and irregularly. CIF improved the damage of endothelium, inhibited the proliferation of smooth mesangial muscle cells, and reduced the irregular state of cell arrangement. The results showed that CIF had a good effect on vascular endothelial injury and imbalance of the vascular endothelial relaxing factor in L-NNA-induced hypertension model rats.

After confirming the potential antihypertension effect of CIF, we try to focus on the mechanisms of CIF about NO. NO from vascular endothelium plays an important role in maintaining blood pressure balance, and its deficiency can lead to a series of cardiovascular diseases [55, 56] such as hypertension and atherosclerosis [57]. It is reported that NO can regulate the expression and activation of adhesion molecules and participate in the prevention and treatment of atherosclerosis, endothelial activation, and T cell infiltration. Nitric oxide synthase (NOS) inhibitors can reduce various vasodilator factors [58–61] and induce endothelium-dependent vasodilation response. Large doses of NOS inhibitors can significantly reduce the production of NO in vascular endothelium in a short time, promote the proliferation of VSMC, and alter vascular tissue structure [62, 63]. This process can damage vascular endothelial function, thereby further restricting the synthesis of NO in endothelial cells to cause and maintain hypertension.

**Figure 14:**
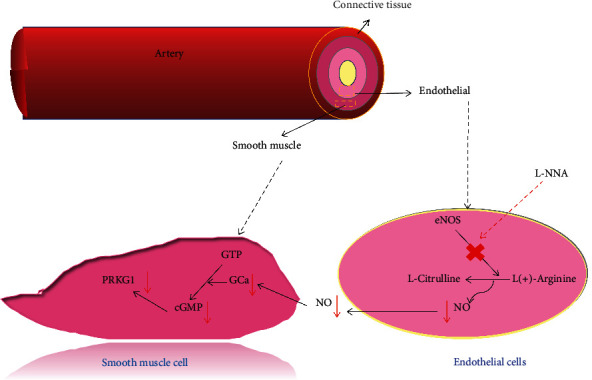
The NO pathway involved in this study is that L (+) arginine oxidizes to NO and L-guanidine. NO activates soluble guanylate cyclase (sGC) by retrograde diffusion, enhances the level of cGMP in cells, and cGMP reregulates cGMP-dependent protein kinase (PRKG1) to reduce intracellular Ca^2+^ and induce vasodilation. L-NNA is used to block NO synthesis and contraction of smooth muscle. “×,” inhibit.

NOS can be divided into three subtypes: neuronal nitric oxide synthase (nNOS/NOS1), endothelial nitric oxide synthase (eNOS/NOS3), and inducible nitric oxide synthase (iNOS/NOS2). The main function of NOS3 is to produce NO in the blood vessel and then regulate vascular tension by inhibiting smooth muscle contraction and platelet aggregation [64–66]. The downstream of the NO signaling pathway is mainly composed of the interaction between NO and heme-containing proteins, the most important of which is guanylate cyclase (GC) [67]. GC is a deterministic hemoprotein formed by the combination of subunit alpha 1 or alpha 2 with common subunit beta 1. L (+) arginine synthesizes NO via NOS3 and produces secondary messenger cGMP by activating soluble GC (sGC) in VSMC, which acts on smooth muscle cells to induce vasodilation and inhibit cell proliferation, reduce intracellular Ca^2+^, and cause vasodilation. PRKG1, a cGMP-dependent protein kinase, is involved in the regulation of smooth muscle relaxation, platelet function, cell division, and nucleic acid synthesis. The NOS3/sGC/cGMP/PRKG1 pathway is particularly important in cardiovascular and nervous systems. It can regulate smooth muscle relaxation, synaptic transmission, and adhesion of endothelial cells, blood cells, and epithelial cells [68–70]. CIF relieves the blood pressure of model rats, which may be affected by the NO pathway, thereby further improving the activation of vasoconstrictor factors (Figure 14).

In this study, we evaluated the blood pressure lowering and blood vessel effects of CIF. The results showed that CIF could reduce the blood pressure of L-NNA-induced hypertension model rats, decrease the levels of ET-1 and TXB_2_ in serum, and increase the levels of NO and PGI_2_. Besides, CIF regulates the NOS3 expression in VECs, increases the NO production, and activates the downstream GCa/cGMP/PRKG1 signaling pathway, thus causing vasodilation. This suggests that CIF could protect endothelial cells from injury and balance the synthesis and release of EDRFs, increase the NOS3 expression in endothelial cells, regulate the synthesis and release of NO, imbalance the EDRFs, and improve the endothelial relaxing and contracting function. Besides, CIF can also improve the pathological morphology of the heart, kidney, and aorta, indicating the advantages of this TCM. Back to the compound target network, luteolin and quercetin were found to influence NOS3 and NOS2 tested targets directly for hypertension, future studies should recognize the role of these compounds in CIF, and pharmacokinetic parameters should be calculated in both healthy and disease.

## 5. Conclusion

Hypertension is a common cardiovascular and cerebrovascular disease affecting people's health. Vascular endothelial function is closely related to hypertension. Vascular endothelial dysfunction plays a significant role in the occurrence and development of hypertension. CIF could regulate the blood pressure of L-NNA-induced hypertension model rats and improve the target organ disease of the heart and kidney. The vascular function might be improved by inhibiting the activation of the NOS3-pathway, providing an experimental basis for the development of hypertension in Chinese medicine.

## Figures and Tables

**Table 1:** Docking results of core components and core targets.

Protein	PDB	Ligand molecule	Actual combined free energy (kcal/mol)
NOS3	3EAH	Luteolin	−9.6
		Quercetin	−9.5
NOS2	3E7G	Luteolin	−10
		Quercetin	−9.7

## Data Availability

The data used to support the findings of this study are available from the Ting Wang upon request.

## Abbreviation

CIF: Yeju Jiangya Decoction
TCM: Traditional Chinese Medicine
KEGG: Encyclopedia of Genes and Genomes
PPI: Protein-protein interaction network
GO: Gene biological process
ET-1: Endothelin-1
TXB_2_: Thromboxane B2
NO: Nitric oxide
PGI2: Prostacyclin 2
WB: Western blot
IHC: Immunohistochemistry
NOS3: Nitric oxide synthase 3
NOS2: Nitric oxide synthase 2
GC-*α*1: Guanylyl Cyclase-alpha 1
GC-*α*2: Guanylyl Cyclase-alpha 2
PRKG1: Protein kinase CGMP-dependent 1
L-NNA: N-Nitro-L-arginine
H&E: Hematoxylin and eosin
eNOS/NOS3: Endothelial nitric oxide synthase
cGMP: Guanosine 3′, 5′-cyclic monophosphate.
